# Supplementary material for: Investigating molecular mechanisms of 2A-stimulated ribosomal pausing and frameshifting in Theilovirus
Source: Nucleic Acids Res. 2021 Nov 9;49(20):11938–58. doi: 10.1093/nar/gkab969 (PMC8599813; doi:10.1093/nar/gkab969)
Supplement: gkab969_Supplemental_File [file gkab969_supplemental_file.pdf]

Supplementary Figures and Legends

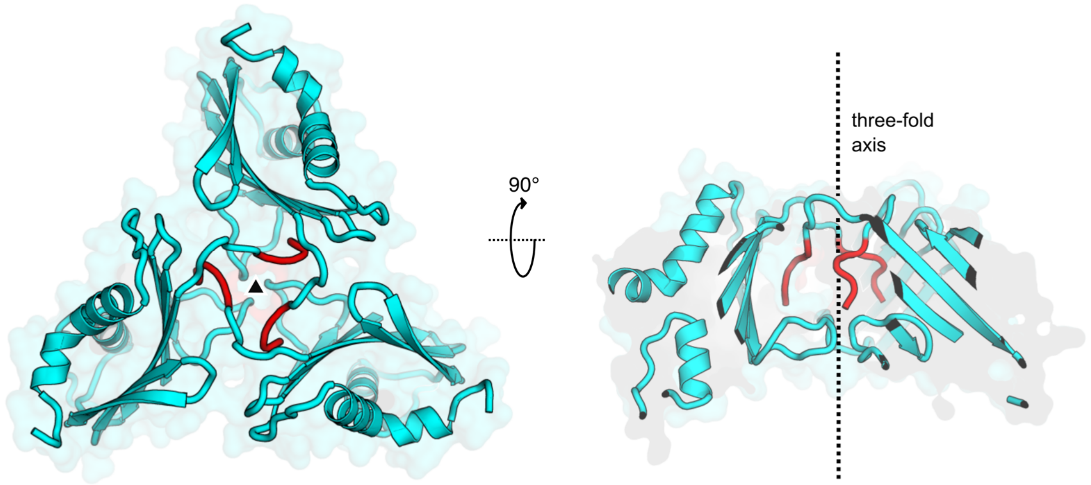

Figure S1. TMEV 2A trimers observed in the crystalline lattice.

View of the trimeric protein assembly at the three-fold crystallographic symmetry axis. TMEV 2A molecules are shown as cartoons (cyan) in two orthogonal views, centred around the symmetry axis (black triangle and dotted line). This arrangement may correspond to trimeric assemblies predicted by PDBePISA (46) and observed as a minor species in solution by SEC-MALS (**Figure 1B**). The interface between subunits is predominantly formed by the N-terminal cloning tag extension ($G_{-4}P_{-3}L_{-2}G_{-1}S_0$; red). This particular trimeric assembly is therefore not likely to be physiologically relevant.

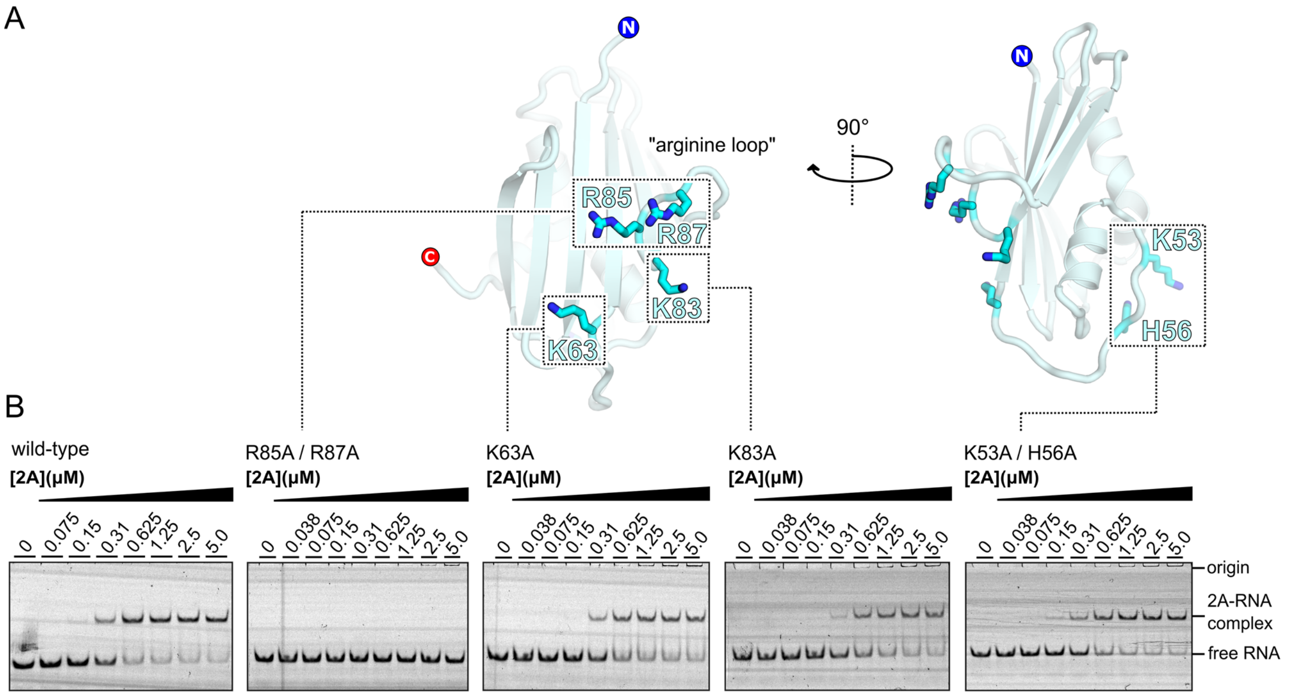

Figure S2. Mutagenesis of putative RNA-binding residues demonstrate the importance of the conserved arginine loop.

A. Mutagenesis of TMEV residues equivalent to those observed at the RNA binding surface in the EMCV 2A-70S$_{IC}$ structure - see also **Figure 2B**. The locations of mutations R85A / R87A, K63A, K83A and K53A / H56A are shown as sticks.

B. EMSA analyses showing effects of the above mutations on stimulatory element RNA binding, compared to a wild-type control. Experiments were conducted with 50 nM Cy5-labelled TMEV 6 RNA and 2A concentrations as indicated between zero and 5.0 μM. Following non-denaturing electrophoresis, fluorescence was imaged using a Typhoon scanner.

Cardiovirus A / encephalomyocarditis virus

```
M81861      AAA CAG GUU UUC CAG ACC CAA GGA GCG GCA GUG UCA UCA AUG GCU CAA ACC CUA CUG CCG AAC GAC CUU
M22457      AAG CAG GUU UUU CAG ACC CAG GGA GCG GCA GUG UCA UCA AUG GCU CAA ACC CUA CUG CCG AAC GAC CUA
KP892662    AAG CAG UUU UUU CAG ACA CAA GGA GCG GCA GUG UCA GCA AUG ACU CAA ACC CUA CUG CCG AGC GAC CUA
LC585221    AAA CAU UUU UUC CAG ACA CAA GGA GCG GCA GUG UCA GCA AUG ACC CAA ACC CUA CUG CCG AAC GAU CUU
KC310737    AAA CAG GUU UUU CAG ACA CAA GGA GCG GCG CUG ACC ACC AUG GCC AAU ACC CUG GCG CCG AGC AAC AUU
JX257003    AAA CAG GUU UUU CAG ACC CAA GGA GCG GCA CUG UCC UCA AUG GUC AAC ACC CUG GUG CCG AAC AAC CUA
```

Cardiovirus B / TMEV, RTV, etc

```
EU542581    CAG UCG GUU UUU CGG CCA CAA GGU GGG GUG CUU ACU AAA UCC CAA GCA CCC AUG UCA GGA AUU CAG AAC
M20301      CAG UCG GUU UUU CAG CCA CAA GGU GCG GUG CUA ACU AAA UCC CUA GCA CCC CAG GCA GGA AUC CAA AAU
MF172923    CAU UCG GUU UUU CAG CCU CAA GGU GCG GCA CUA ACU AAG UCC CUA GUG CCU CAG GCA GGA AUU CAG AAU
MF352420    AUG UCG GUU UUU CAG CCU CAA GGU GCG GCG CUA ACU AAG UCC CUA GCG CCA CAG ACA GGG AUU CAG GGA
```

Cardiovirus D / Saffold virus

```
EU376394    CAG UCG GUU UUU CAG UUG CAA GGU GGG GUG CUA ACU AAA UCC CAA GCA CCC AUG UCU GGU UUA CAG AGU
```

Cardiovirus E, *F* and unassigned

```
KY432928    AUG UCG GUU UUU CAG CCA CAA GGU GGU GUG CUC ACU AAA ACC CGA GCA UAC GCA GAU AAU GUG AAG GGA
KY432930    GCU CAG GUU UUU CAG GAA CAA GGC GCG GUA CUG ACU AAA UCC CCA GUA CCU ACA AUU CAA AGU UUU GUA
KY855434    AUG UCG GUU UUU CAG GAA CAA GGC GCG GUG CUA ACU AAA GUC CCU GCA CCG AAA GAU GUU UUG UCA AAC
KF823815    AAC CAG GUU UUU CAG CCA CAA GGA GCG GCU CUA ACU AAA GCC CAA GAG CCG GCA CAA GAU AUG GUA AAG
                shift site                  stem-loop
```

Figure S3. Conservation at the frameshift stimulatory site in cardioviruses.

Sequence of the PRF region from representative cardiovirus isolates, showing the highly conserved shift site (light blue) and 3′ RNA stem-loop structure (green). Nucleotide variations in the shift site are indicated in pale pink. Paired substitutions that preserve the predicted structure are highlighted in crimson, purple or orange; single substitutions that – via G:U base-pairing are compatible with the predicted structure – are highlighted in cyan. The conserved CCC loop triplet is shown in blue.

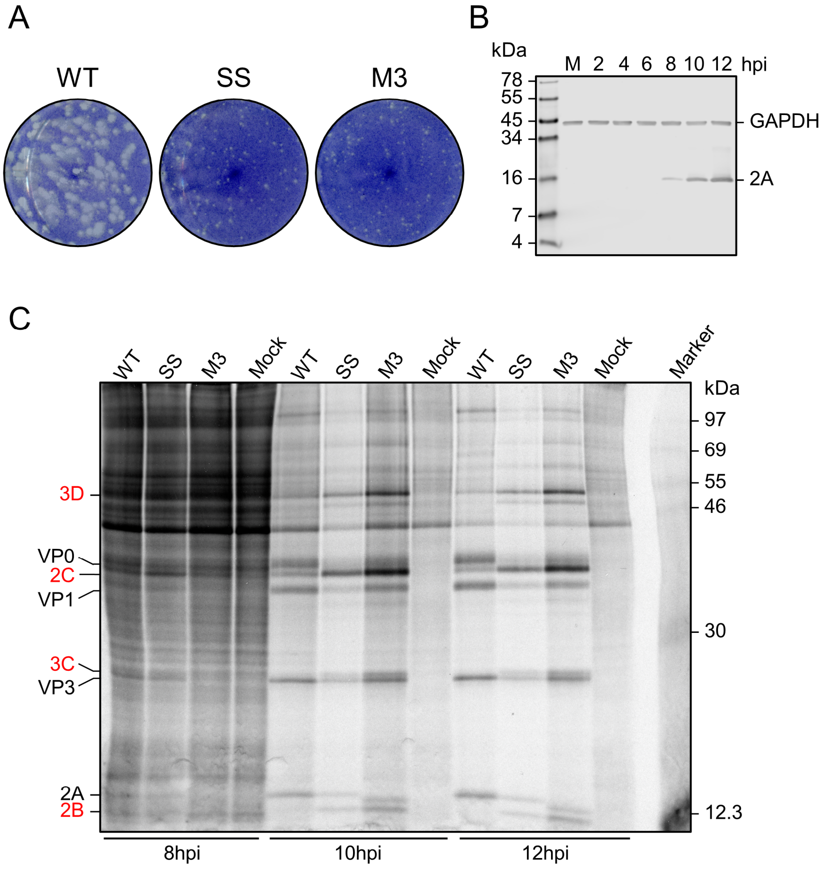

Figure S4. WT and mutant 2A in the context of infection.

A. Plaque assays of BSR cells infected with WT, SS, or M3 TMEV and fixed at 48 hpi.

B. Western blot of BSR cells infected with WT TMEV and harvested over a timecourse up to 12 hpi, or mock-infected (M) and harvested immediately. The SS mutant was assayed in parallel and produced similar results (data not shown).

C. Metabolic labelling of BSR cells infected with WT, SS, or M3 TMEV and harvested over a timecourse of 8—12 hpi. Positions of TMEV proteins are indicated, with those downstream of the frameshift site written in red. At 8 hpi, reliable quantification of viral proteins above the background of host translation was not possible. However, at 10 and 12 hpi, a large proportion of ongoing translation is viral, likely due to virus-induced shut-off of host gene expression, and viral proteins were clearly visible and quantifiable.

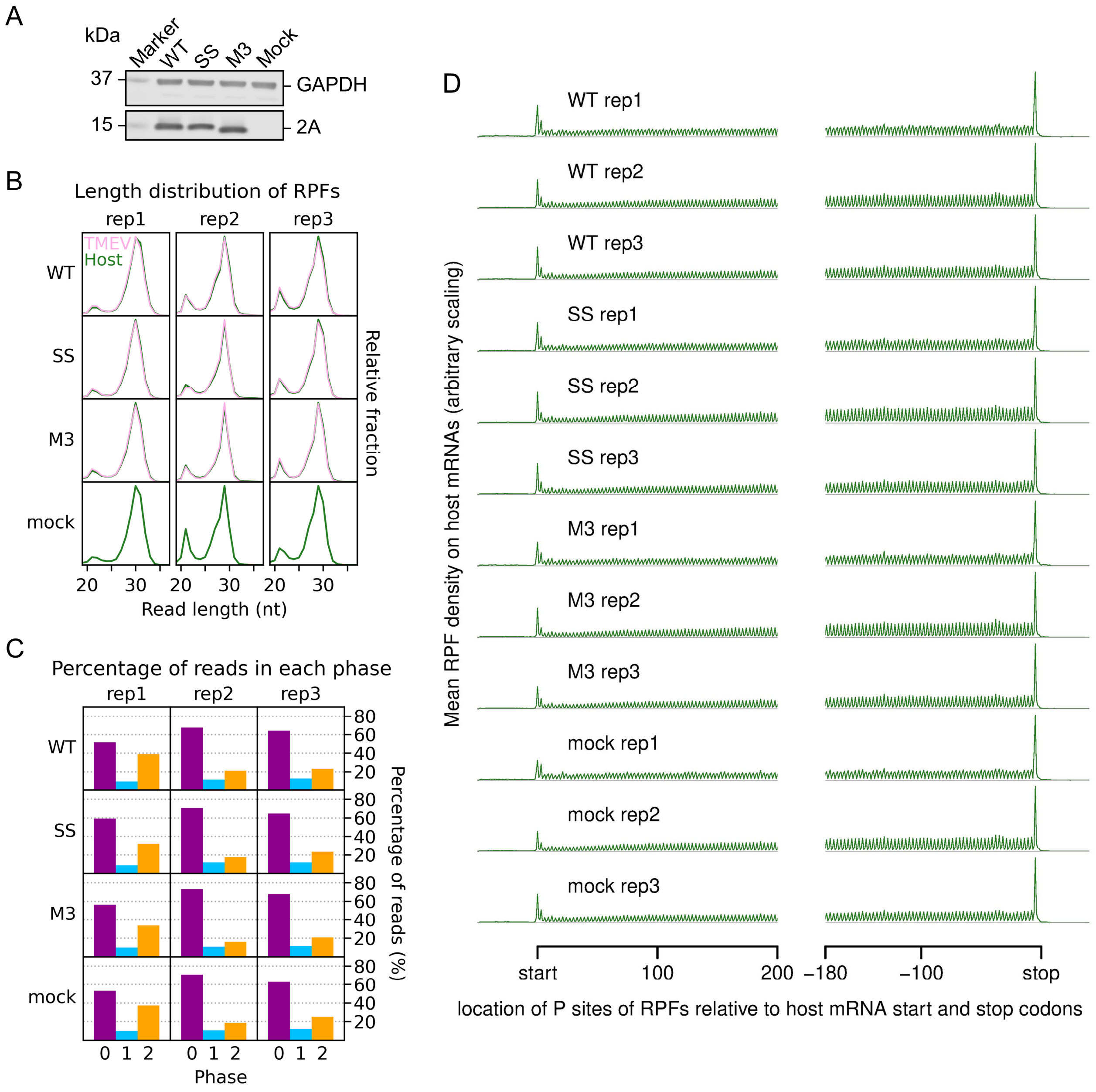

Figure S5. Ribosome profiling quality control analysis indicates high quality data.

A. Western blot of lysates used for ribosome profiling. Note that the mutant M3 2A migrates slightly faster during gel electrophoresis than the WT protein.

B. Length distribution of RPFs mapping within host (green) and viral (pink, mock excluded) CDSs in each library.

C. Percentage of reads (all read lengths) attributed to each phase, from reads mapping within host CDSs. Noting that approximately one third of the reads in replicate 1 libraries were attributed to the –1/+2 phase, we increased the amount of RNase I added to further replicates to improve trimming, yielding the very high proportion of phase 0 reads seen in replicates 2 and 3. Phase compositions of virus CDS-mapping reads closely matched those of host-mapping reads (**Figure 7A**, upstream and downstream panels).

D. Metagene profile of the distribution of inferred P site positions of host mRNA-mapping reads relative to start and stop codons. Only transcripts with an annotated CDS of at least 150 codons and UTRs of at least 60 nt were included in the analysis, and the total number of reads from all these transcripts mapping to each position was plotted. RPFs map to coding sequences with a triplet periodicity and few RPFs map to the UTRs, particularly the 3′UTR. Typically heightened RPF peaks corresponding to the sites of translation initiation and termination are also observed (55,87).

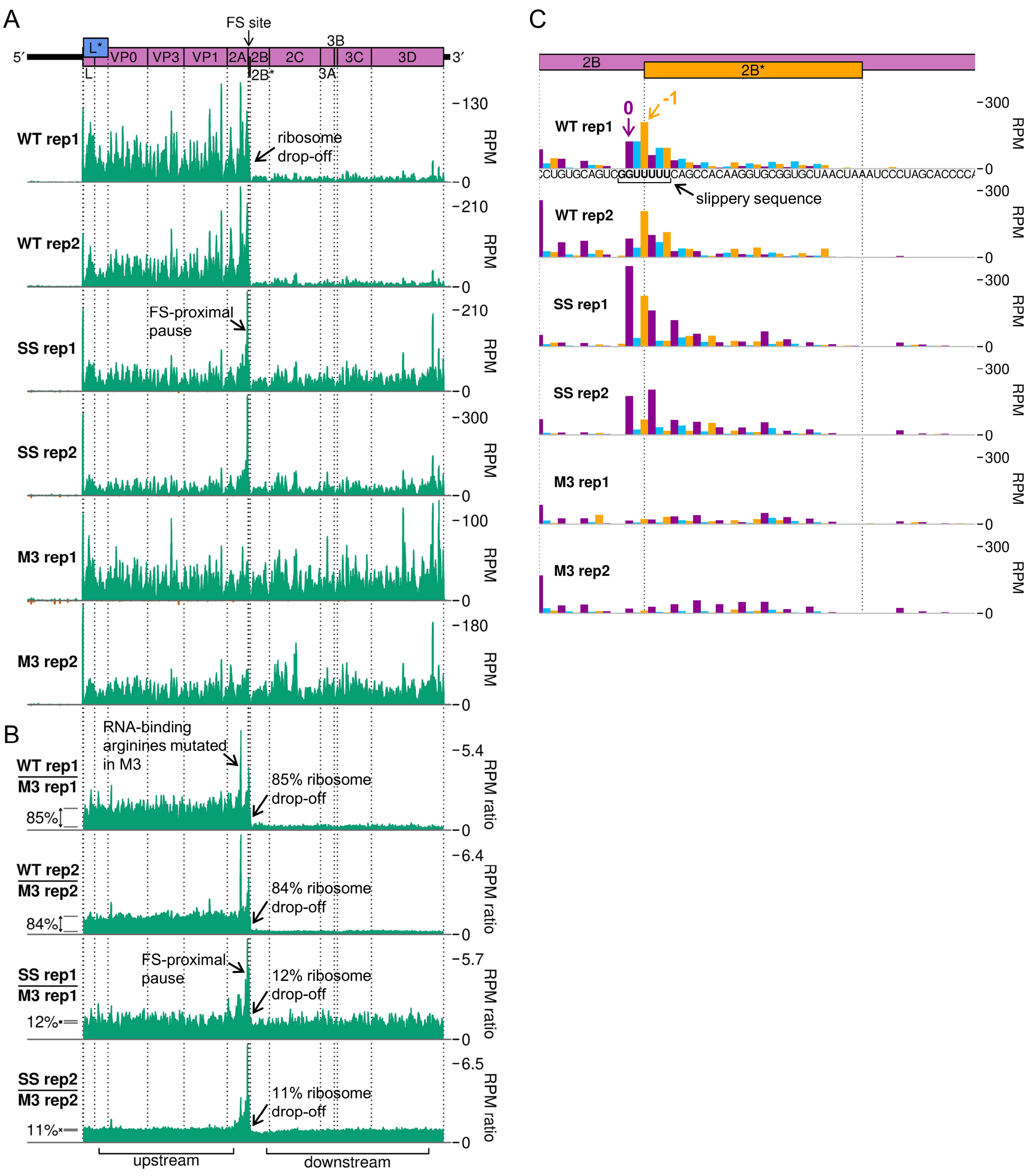

Figure S6. Further replicates of ribosome profiling RPF distribution plots.

A. RPF distribution on the viral genome, as in main text Figure 6D, for replicates 1 and 2.

B. Ratio of RPF density on the WT or SS genome normalised by M3, as in main text Figure 6F, for replicates 1 and 2.

C. RPF distribution at the frameshift site, as in main text Figure 6G, for replicates 1 and 2.

Supplementary Tables

Table S1 – Crystallographic data collection and refinement.

	Highest resolution†	Structure determination†
Data collection*		
Wavelength (Å)	0.9159	0.9159
Space group	$P\,2_1\,3$	$P\,2_1\,3$
Cell dimensions		
a = *b* = *c* (Å)	73.77	74.24
Resolution (Å)	43.59—1.87 (1.92—1.87)	52.48—2.11 (2.15—2.11)
Unique reflections	11,355 (577)	8100 (389)
Completeness (%)	100.0 (100.0)	100.0 (100.0)
Anomalous	-	100.0 (100.0)
Multiplicity	19.0 (19.8)	19.4 (20.3)
Anomalous		10.3 (10.6)
R_{merge}	0.113 (1.191)	0.078 (1.956)
R_{pim}	0.026 (0.273)	0.025 (0.629)
$CC_{1/2}$	0.999 (0.644)	1.00 (0.715)
CC_{anom}	-	0.504 (0.002)
Mean I/σ(I)	16.8 (2.8)	26.9 (1.7)
Wilson B (A^2)	26.06	46.96
Refinement*		
Resolution (Å)	42.61—1.87 (1.93—1.87)	
Reflections ‡	21,277 (2095)	
Working set	20,162 (1961)	
Test set	1115 (134)	
R_{work}	0.1847	
R_{free}	0.2302	
No. of atoms		
Protein	1112	
Solvent	98	
Ions	1	
Root mean square deviation		
Bond lengths (Å)	0.005	
Bond angles (°)	0.890	
Ramachandran favoured (%)	100	
Ramachandran outliers (%)	0.0	
Poor rotamers (%)	0.0	
Mean B value (A^2)	31.6	

*Values in parentheses are for the highest resolution shell
† Both crystals were harvested from the same crystallisation drop
‡ I+ and I- were treated as separate reflections in phenix.refine

Table S2. Number of ribosome profiling reads assigned to each category. The "_broad" samples are the broad-spectrum libraries, for which 35—65 nt fragments were purified. Reads under 19 nt long (for monosome libraries) or under 35 nt long (for broad-spectrum libraries) were defined as too short. Some categories are not applicable to the broad-spectrum libraries due to minor differences in computational processing (detailed in Methods).

Virus	Replicate	Total reads	Too short	Adapter-only	No adapter	PCR duplicates	rRNA
WT	1	1790088	61648	876	12076	28721	586963
WT	2	26408581	2340239	297018	116064	1834921	2926112
WT	3	28688961	2756571	280096	406493	2627172	4719318
SS	1	2392994	34429	1859	9565	61705	505571
SS	2	25027728	2854707	353732	126430	1456929	2342432
SS	3	21788351	1927596	320336	503529	2258152	3634096
M3	1	2290607	149115	6052	18977	61127	619963
M3	2	27308973	1702862	157048	138108	1654696	2986823
M3	3	30504465	2009396	515817	438248	3248459	5122599
Mock	1	1914259	171017	1448	27268	34259	495928
Mock	2	26398293	2345528	117083	120712	1286218	2852963
Mock	3	27548554	1532098	145612	608503	2112106	4820304
WT_broad	3	10624342	2528401	1155			2546503
M3_broad	3	7230428	1686167	1182			2090553
mock_broad	3	9730736	1814114	950			3247839

Virus	Replicate	vRNA	mRNA	ncRNA	mtDNA	gDNA	BL21 E. coli
WT	1	60322	371302	71085	1909	81527	2196
WT	2	789308	3476556	1470387	119371	930513	8738367
WT	3	1299982	5671747	2271869	61429	1890651	914704
SS	1	198472	700308	189411	7642	139138	6991
SS	2	769385	2293504	980217	49001	527344	11085126
SS	3	1136138	4143979	1661076	64024	1486102	650083
M3	1	102620	485732	111611	2746	129914	5070
M3	2	946929	3136082	1210389	56677	837885	12118144
M3	3	1669867	6825341	2494147	105091	2404980	586000
Mock	1	161	481985	88888	3701	105409	4205
Mock	2	426	4817616	904360	26897	1216025	10404660
Mock	3	742	9839798	1651028	67215	1765457	427314
WT_broad	3	97581	534229	1322626	271988	413111	485534
M3_broad	3	20310	327007	783510	268166	270980	110028
mock_broad	3	31	349897	1038588	294835	295848	163860

Table S3. Phosphorimager signals for viral-specific bands on metabolic labelling gels.

<u>10 hpi replicate 1</u>

Protein	WT	SS	M3	Carried forward for calculations?
3D	2395.08	2470.9	6697.62	Yes
VP0	3099.31			No - band too faint for reliable quantification in mutant lanes
2C	2741.89	5435.87	10800.7	Yes
VP1	2976.72	1511.32	3126.03	Yes
3C		861.87	1954.94	No - band too faint for reliable quantification in mutant lanes
VP3	2703.24	1402.52	3178.16	Yes
2A	2325.6	1083.08	1736.97	No - methionine-normalised results suggest difference in turnover rate between WT and mutant 2A
2B	853.34	1141.5	2323.76	Yes

<u>10 hpi replicate 2</u>

Protein	WT	SS	M3	Carried forward for calculations?
3D	28715.83	26525.91	67503.74	Yes
VP0	32548			No - band too faint for reliable quantification in mutant lanes
2C	25909.88	39483.35	90913.71	Yes
VP1	28175.9	13058.42	31910.11	Yes
3C		9944.51	20698.75	No - band too faint for reliable quantification in WT lane
VP3	23789.64	12568.31	28983.54	Yes
2A	15459.04	8312.66	13068.13	No - methionine-normalised results suggest difference in turnover rate between WT and mutant 2A
2B		9735.73	16639.4	No - band too faint for reliable quantification in WT lane

<u>10 hpi replicate 3</u>

Protein	WT	SS	M3	Carried forward for calculations?
3D	1272.31	1287.21	2652.87	Yes
VP0	1577.01			No - band too faint for reliable quantification in mutant lanes
2C	1116.87	2546.23	3469.65	Yes
VP1	1155.83	729.01	889.83	Yes
3C			600.71	No - band too faint for reliable quantification in WT lane
VP3	1089.86	608.08	1074.88	Yes
2A				No - band not resolvable from dye front
2B				No - band not resolvable from dye front

12 hpi replicate 1

Protein	WT	SS	M3	Carried forward for calculations?
3D	14521.16	21433.11	43338.38	Yes
VP0	31017.26			No - band too faint for reliable quantification in mutant lanes
2C	19216.2	33218.68	63307.42	Yes
VP1	28838.43	11491.95	20683.21	Yes
3C		9132.88	16079.93	No - band too faint for reliable quantification in WT lane
VP3	23912.68	11430.74	20631.82	Yes
2A	15548.96	7338.38	8650.82	No - methionine-normalised results suggest difference in turnover rate between WT and mutant 2A
2B	5747.61	7299.89	11206.57	Yes

12 hpi replicate 2

Protein	WT	SS	M3	Carried forward for calculations?
3D	945.31	2963.3	7539.19	Yes
VP0	2397.33			No - band too faint for reliable quantification in mutant lanes
2C	1470.08	5552.5	10498.07	Yes
VP1	2248.87	1656.4	3280.92	Yes
3C		963.09	1771.51	No - band too faint for reliable quantification in WT lane
VP3	2434.33	1869.35	3682.06	Yes
2A	1847.17	1285.68	1844.53	No - methionine-normalised results suggest difference in turnover rate between WT and mutant 2A
2B	476.88	1350.26	2322.62	Yes

12 hpi replicate 3

Protein	WT	SS	M3	Carried forward for calculations?
3D	664.88	7645.55	8779.04	Yes
VP0	1760.34			No - band too faint for reliable quantification in mutant lanes
2C	934.12	7949.59	10576.14	Yes
VP1	1515.47	2134.25	2391.4	Yes
3C		952.4	621.45	No - band too faint for reliable quantification in WT lane
VP3	1619.9	2328.56	2969.14	Yes
2A				No - band not resolvable from dye front
2B				No - band not resolvable from dye front
